# The phycobilisomes: an early requisite for efficient photosynthesis in cyanobacteria

**DOI:** 10.17179/excli2014-723

**Published:** 2015-02-20

**Authors:** Niraj Kumar Singh, Ravi Raghav Sonani, Rajesh Prasad Rastogi, Datta Madamwar

**Affiliations:** 1 Shri A. N. Patel PG Institute (M. B. Patel Science College Campus), Anand, Sardargunj, Anand - 388001, Gujarat, India; 2 BRD School of Biosciences, Sardar Patel Maidan, Vadtal Road, Post Box No. 39, Sardar Patel University, Vallabh Vidyanagar 388 120, Anand, Gujarat, India

**Keywords:** protein, phycobiliproteins, chromophores, linker polypeptide, complementary chromatic adaptation

## Abstract

Cyanobacteria trap light energy by arrays of pigment molecules termed “phycobilisomes (PBSs)”, organized proximal to "reaction centers" at which chlorophyll perform the energy transduction steps with highest quantum efficiency. PBSs, composed of sequential assembly of various chromophorylated phycobiliproteins (PBPs), as well as nonchromophoric, basic and hydrophobic polypeptides called linkers. Atomic resolution structure of PBP is a heterodimer of two structurally related polypeptides but distinct specialised polypeptides- a and ß, made up of seven alpha-helices each which played a crucial step in evolution of PBPs. PBPs carry out various light dependent responses such as complementary chromatic adaptation. The aim of this review is to summarize and discuss the recent progress in this field and to highlight the new and the questions that remain unresolved.

## Abbreviations

PBS – Phycobilisome; PBP – Phycobiliprotein; LHC – Light harvesting complex; PE – Phycoerythrin; PC – Phycocyanin; AP – Allophycocyanin; F-αCPE – Fragmented α PE; PEB – Phycoerythrobilin; PCB – Phycocyanobilin; PUB – Phycourobilin; PVB – Phycoviolobilin; RC – Reaction center; CCA - Comple-mentary chromatic adaptation

## Introduction

Cyanobacteria are the oldest group of oxygenic prokaryotes in the history of photosynthetic life having cosmopolitan distribution in both aquatic and terrestrial ecosystems (Whitton, 2012[100]). They are most important component of photoautotrophic microflora in terms of total biomass and productivity, maintaining the trophic energy dynamics of an ecosystem (Iturriaga and Mitchell, 1986[35]; Stock et al., 2014[92]). They are credited for the evolution of existing aerobic life on the Earth’s surface due to their inherent capacity of photosynthesis mediated oxygen evolution (Olson, 2006[69]). Adaptive diversification of cyanophyceae to optimize their oxygenic photosynthesis in a range of ecological niches over the last 3.5 billion years (Fischer, 2008[23]; Feyziyev, 2010[21]) has led to the evolution of a large and diverse array of photosynthetic pigments, each with specialized functions to compete them successfully on the planet.

The oxygenic photosynthetic organisms such as cyanobacteria contain two distinct reaction centers (RCs) i.e., P700 and P680 of photosystem I (PSI) and photosystem II (PSII), respectively. Each RC is associated with an antenna of light-harvesting protein-pigment complexes (LHC) (Bearden and Malkin, 1975[9]). Chlorophyll *a *(Chl *a*) is the universal reaction center pigment biomolecule in oxygen-evolving organisms. In higher plants and green algae, the major LHC consists of membrane associated Chl *α/b*-binding (Cab) proteins which are integral membrane proteins, arranged in trimeric arrays (Kuhlbrandt and Wang, 1991[51]). In marine algae such as diatoms, dinoflagellates, brown algae, and chrysophytes, the major light-harvesting complex contains xanthophylls, such as fucoxanthin or peridinin, Chl *a* and Chl *c*. The LHC of cyanobacteria, eukaryotic red algae, cryptophytes and glaucophytes consist of Chl *a* and phycobilisomes (PBSs). The PBSs are light harvesting antennae of cyanobacterial photosystem which consist of key photosynthetic pigment molecules, the phycobiliproteins (PBPs). These intensely colored biliproteins are homogenous family of light harvesting proteins which absorb visible light in the range of 450 to 670 nm. They transfer excitation energy with high quantum efficiency to photosystem II and I in the photosynthetic lamellae (MacColl, 1998[62]). Quality of incident radiation and efficient energy transfer involved in light harvesting evolve PBSs to gain the ability to survive in broad range of environments including different depths of sea and fresh water. Many cyanobacteria have shown a tendency to respond to a change in light quality by changing the composition of their antenna in such a manner that it increases the proportion of the pigment with absorption spectrum more nearly complementary to the available light (Kehoe, 2010[42]).

### Phycobilisomes

The PBSs are supramolecular complex composed of a core substructure and peripheral rods, and account for up to 60 % of the total protein mass in cyanobacteria. The molecular weight of PBS varies from 7 to 15 MDa which is far larger than the reaction centers i.e., PSI and PSII (Mullineaux, 2008[66]) and utilizes 30-50 % of the total light-harvesting capacity of cyanobacterial and red algal cells and transfers the energy to reaction centers (Wang et al., 1977[99]). Apart from light absorption and transduction, an additional function of the PBS so far identified is as an emergency source of nutrients in case of nitrogen, sulfur or carbon starvation (Parmar et al., 2011[72]). Ordered disassembly of the PBS complex during starvation requires the presence of the products of a number of non-bleaching (*nbl*) genes (Grossman et al., 2001[30]). However, different disassembly pathways may occur in different organisms. Soni et al. (2010[90]) identified a 14 kDa truncated fragment of α-subunit of phycoerythrin (F-αPE) from the degraded and stored samples of 19 kDa protein of C-phycoerythrin (C-PE). Even so, the specific mechanism of nbl-A or nbl-B gene products is still unclear (Baier et al., 2001[8]).

#### Phycobilisome morphology

**Figure 1:**
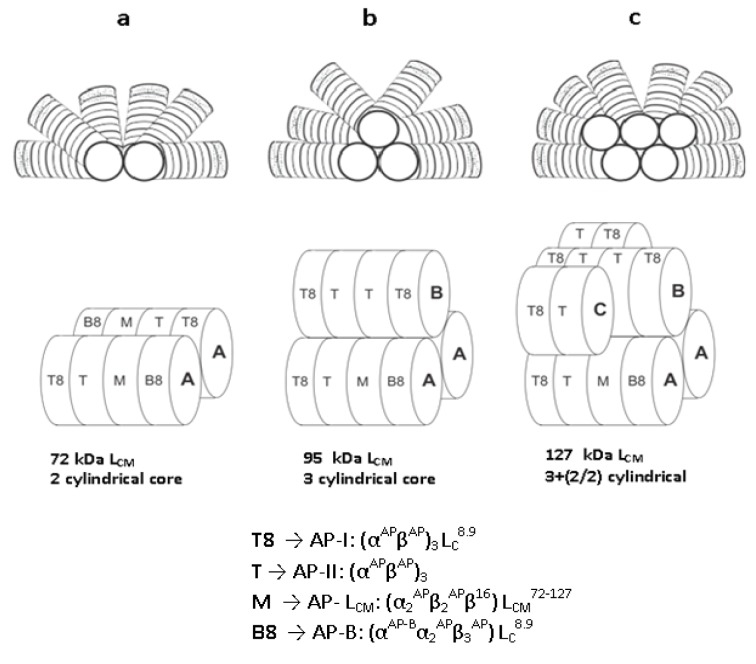
Schematic representation of the three types of hemi-discoidal PBS. a, PBS with bicylindrical core. Bicylindrical core consists of two identical asymmetric cylinders arranged in anti-parallel manner, which is made up of four allophycocyanin trimer disk, arranged in sequence of T8-T-M-B8; b, PBS with tricylindrical core. Tricylindrical core consists of two types (asymmetric T8-T-M-B8 and symmetric T8-T-T-T8) of allophycocyanin cylinders. Two similar types of cylinders are arranged in same manner as in bicylindrical core, whereas third symmetric cylinder (T8-T-T-T8) is arranged on them as shown in figure; c. PBS with pentacylindrical core. Pentacylindrical core contains two extra asymmetric half-cylinders (only two trimer disks- T-T8) beside the symmetric cylinder as shown in figure, oriented in anti-parallel manner to each other. T8-disk is made up of α- and ß-subunits of allophycocyanin and core linker peptide (L_C_^8.9^); T-disk contains an α- and ß-subunit of allophycocyanin without any linker peptide; M-disk is composed of α-, ß- and variant ß-(16 kDa)subunits of allophycocyanin and core-membrane linker peptide (L_CM_^72-127^). B8-disk comprises of α-, variant α- and ß-subunits of allophycocyanin and core linker peptide (L_C_^8.9^).

**Figure 2:**
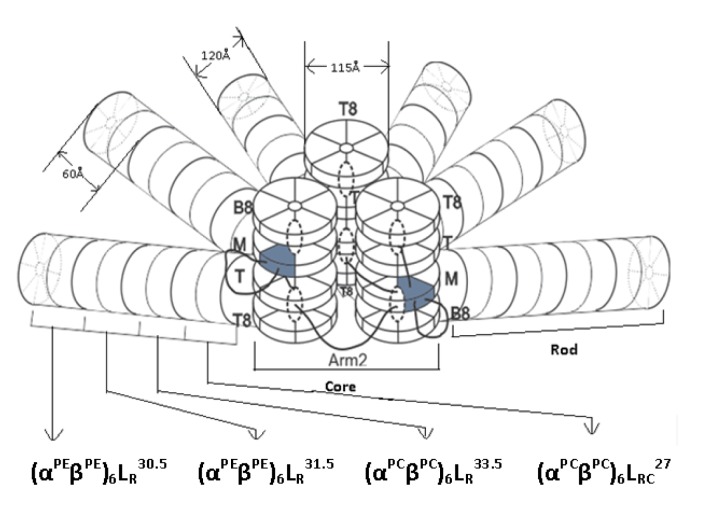
Structural model of a hemi-discoidal PBS, as seen from the thylakoid membrane upwards. The architecture exhibits a tri-cylindrical core, from which six rods composed of PC and PE hexamers radiate outwards.

The structure and size of PBSs vary widely. Three classes of structures have been described to date: (a) hemi-discoidal phycobilisomes; so far seen in red algae, cyanobacteria, and certain cyanelles; (b) "rod bundle" shaped phycobilisomes - represented until now only by those of the thylakoid-less cyanobacterium *Gloeobacter violaceus* (Gysi and Zuber, 1974[32]) and (c) hemi-ellipsoidal; so far seen only in red algae. Hemi-ellipsoidal phycobilisomes have molecular weights of 20 MDa, whereas others are in range of 5-8 MDa (MacColl, 1998[60]). The family of extensively found hemidiscoidal PBSs has been further divided into three sub-groups according to structural differences of the core domain (Sidler, 1994[82]). This domain is composed of 2, 3 or 5 cylinders (Figure 1) of stacked allophycocyanin trimers (α_3_ß_3_), with a diameter of about 110-117 Å of each and its length is given by the stacking of two to four discs of thickness ~30 Å each (Arteni et al., 2009[5]). Each of the cylinders belonging to the core substructure is composed of four trimeric allophycocyanin (AP) discs. However, pentacylindrical core contains two extra cylinders having only two discs (Figure 1(Fig. 1)). A series of 6 to 8 rods radiate from the core which is composed of cylinders of stacked hexameric (α_3_ß_3_)_2_ discs. Each rod consists of two to four discs having diameter 110-120 Å and thickness 60 Å each (Adir, 2005[1]) (Figure 2(Fig. 2)). 

The rods from various organisms are quite variable in the composition with number of discs and the ratio of PE to PC, depending on the growth environment (Lüder et al., 2001[57], 2002[56]). Single particle microscopy studies showed that the interaction between AP trimer is looser than that of PC trimmers forming hexamers (Arteni et al., 2009[5]). The PBSs containing tri-cylindrical cores are widely spread in cyanobacteria such as *Synechocystis* sp. PCC6803 (Redecker et al., 1993[74]). Tri-cylindrical cores consist of two anti-parallel basal cylinders and one upper cylinder with internal two-fold symmetry (Arteni et al., 2009[5]) (Figure 1b(Fig. 1)). Whereas bi-cylindrical cores contain only cylinders arranged anti-parallel to each other (Lundell and Glazer, 1983[58]) (Figure 1a(Fig. 1)). The penta-cylindrical cores consist of two anti-parallel basal cylinders, one upper cylinder with internal two-fold symmetry and two anti-parallel additional cylinders. These types of cores present in *Mastigocladus laminosus* and *Anabaena* sp. PCC7120, Synechoccous sp PCC7942 (Kana et al., 2009[40]) are less common (Figure 1c(Fig. 1)). Due to lack of integral membrane domain in PBS, it diffuses through the cytoplasm and interacts with thylakoid membrane. Diffusion reflects fluid nature of PBS, which is influenced by their size but not by temperature (Kana, 2013[38]).

### Phycobiliproteins

PBSs are composed of chromophore-associated water soluble acidic polypeptides called PBPs and nonchromophoric basic polypeptides called linker proteins that are hydrophobic. PBPs function as the principal photoreceptor in cyanobacteria and some other algae (Chaiklahan et al., 2011[13]). PBPs are linked to characteristic tetrapyrrole chromophores called bilins, which are covalently bound to the apoprotein via one or two thioether bonds (Scheer and Zhao, 2008[80]). PBPs may comprise more than 60 % of the total soluble cellular protein fraction, which is equivalent to almost 20 % of the total dry weight of cyanobacteria (Soni et al., 2008[91]). The assembly of PBS is initiated with chromophorylation of PBPs, followed by methylation and oligomerization, and linker protein insertion (Miller et al., 2008[63]). PBS assembly and architecture are driven to facilitate energy transfer from higher to lower energy state. 

**Figure 3:**
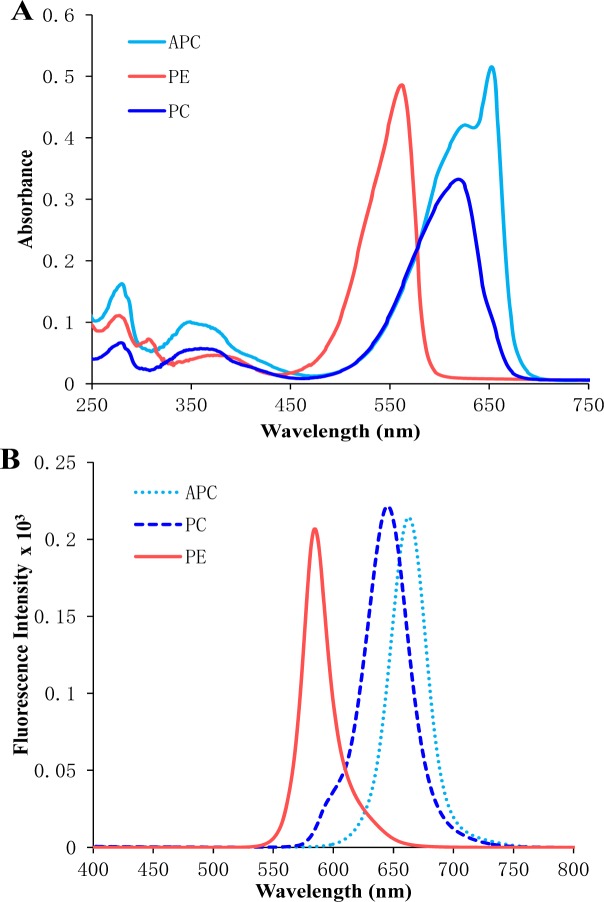
UV-visible (A) and fluorescence spectra (B) of purified phycoerythrin, phycocyanin, allophycocyanin isolated from *Lyngbya* sp. A09DM. Phycoerythrin, phycocyanin and allophycocyanin were exited at 559, 589, 645 nm respectively, to measure their fluorescence emission spectra.

On a functional level, PBPs are a group of intensely colored proteins that occur in cyanobacteria and other algae. Based on their colour, absorption maxima and bilin energy PBPs can be sub-divided into three main groups i.e., (1) High energy-phycoerythrin (PE – bright pink - λmax 540-570 nm), (2) intermediate energy-phycocyanin (PC – dark cobalt blue – λmax 610-620 nm), and (3) low energy-allophycocyanin (AP – brighter aqua blue - λmax 650-655 nm) (Moraes and Kalil, 2009[64]; Sonani et al., 2014[89],[88] ) (Figure 3(Fig. 3)). The visible absorption spectra of the biliproteins are determined by the open-chain tetra-pyrrole chromophore present and by the environment in which they are located. Interaction of chromophore, apoprotein, nonchromophoric linker polypeptide and level of organization makes PBS especially suitable for the energy absorption, transfer and funneling to the chlorophyll-containing reaction centers within the membrane.

PBPs are typically composed of various aggregates of αß where α and ß are homologous polypeptide chains (or ‘subunits’) which are encoded by genes usually present in an operon, with molecular masses in the range of 12–20 and 15–20 kDa, respectively (Singh et al., 2009[83]). The α- and ß-subunits of PBPs are uniform in length with 160 to 180 amino acid residues, respectively. X-ray crystallographic studies have shown that the fold of α and ß subunit of PBPs are a well-defined helical globin-like domain with seven helices (A, B, E, F', F, G and H) and helical hairpin domains (X and Y) at the N-terminus of each chain which are (α/ß) monomers (Kikuchi et al., 2000[45]). These subunits along with bilins, oligomerize themselves to form hexameric and trimeric discs which further participate in the construction of rod and core. The different absorption and emission spectra of the consecutive disc ensures a rapid flow of energy from the donor bilins to the acceptor bilins. Position of PBPs and the influence of the linker polypeptide contribute to rapid directional flow of the excitation energy from one acceptor bilin to the other moving towards the core. The organization of the core is not very well known, but features are similar to those seen in the rods governing the polar transfer of energy. Amino acid sequence data (Zuber, 1983[108]) and DNA sequence data (Bryant et al., 1985[12]) shows considerable conservation of sequence among the various PBPs (AP, PC and PE) as well as between α- and ß-subunits of a given PBP. These findings suggest that PBP genes arose via duplications of a single ancestral sequence. It has also been suggested that an insertion of short DNA-fragments plays a role in the evolution of the ß-subunit ancestral gene from that for α-subunit (Zhao and Qin, 2006[104]). Another way of PBP diversity is due to variation in the structure of the chromophore (bilin) prosthetic groups.

#### Phycoerythrin

Phycoerythrin (PE) occurs as a trimeric (α_3_ß_3_), hexameric (α_3_ß_3_)_2_ and other oligomeric complexes along with chromophores attached to them. Each of α- and ß-subunits of the PBP complex are generally attached to two bilin chromophores. In most PE containing cyanobacterial strains isolated to date, the distal part of the PBP rod is composed of two types of PE (PE I and PE II). PE I binds either only to phycoerythrobilin (PEB) or to both PEB and phycourobilin (PUB) (Amax = 495 nm) whereas, PE II always binds to both PEB and PUB. The PE can be classified into four classes: C-PE (cyanobacterial PE), B-PE (Bangiophyceae PE, containing PEB only), B-PE (Bangiophyceae PE, containing PEB and PUB) and R-PE (Rhodophyta PE), based on their origins and absorption properties (Gantt and Lipschultz, 1974[25]). The C-PE, which also assembles to discs, has a-polypeptide with two chromophore and ß with three (Table 1(Tab. 1)). Pan et al. (1986[70]) have researched the spectral properties of PEs in marine red algae and reported that R-PEs have two spectral types i.e., ‘‘two-peak’ type and “three-peak” type (Pan et al., 1986[70]). Soni et al. (2010[90]), Parmar et al. (2011[72]) and Sonani et al. (2015[87]) have reported the 14 kDa (fragmented-PE, F-PE) and 15.45 kDa (truncated-PE, T-PE) single subunit PE generated due to* in vivo* and *in vitro* truncation. Structural (Soni et al., 2010[90]) as well as denaturation kinetics (Sonani et al., 2015[87]; Anwer et al., 2015[3]) study revealed the maintenance of F-PE and T-PE functionality even after the truncation.

#### Phycocyanin

Phycocyanin is found in complex of trimers (α_3_ß_3_), hexamers (α_3_ß_3_)_2_ and other oligomers plus chromophore attached to them, generally one to α- and two to ß-subunits. Trimers (α_3_ß_3_) are ring-like assemblies of three monomers (αß) with a three-fold symmetry. The hexamers (α_3_ß_3_)_2_ are disc-shaped and formed by face-to-face assembly of trimers. Rods are formed by face-to-face assembly of these discs. In most of the PE containing marine cyanobacterial species characterized so far, PC makes up the basal disc at the core proximal end of the rod. There are many types of phycocyanin depending on the type of attached chromophore, found in cyanobacteria like C-PC (contain only PCB (λmax: 620 nm)), R-PC II (contain both PEB (λmax: 540 nm)) and PCB in the molar ratio of 2:1) and R-PC III (an optically unusual PC that contains both PEB and PCB in the molar ratio of 1:2) (Six et al., 2007[85]) (Table1(Tab. 1)). 

### Allophycocyanin

Allophycocyanin (AP), organized as trimer (α_3_ß_3_) at neutral pH, consists of three ß-polypeptides; each of this polypeptide is associated with one of bilin, which forms the major building blocks of the core. The fluorescence properties of adjacent PC and absorption properties of AP ensure that latter function as an efficient acceptor. Moreover, there are three other core proteins with chromophores, namely L_cm_, ß^16^ and α^B^. The L_cm_ and α^B^ biliproteins receive energy from AP and energy from them is transferred to chlorophyll in the thylakoid membrane. These two biliproteins have emission maxima of about 680 nm, which allows them to transfer energy efficiently to chlorophyll (MacColl, 2004[62]). Cyanobacteria also contain orange carotenoid protein (OCP) to protect photosynthetic system against photo-oxidation (Kirilovsky and Kerfeld, 2012[47]). This process requires the binding of the red active form of the OCP, which can effectively quench the excited state of one of the AP bilins (Tian et al., 2012[97]). However, Jallet et al. (2012[36]) demonstrated that terminal emitters (ApcD, ApcF and ApcE) are not required for the OCP-related fluorescence quenching and they strongly suggested that the site of quenching is one of the AP trimers emitting at 660 nm.

### Evolution of phycobiliproteins

Different components of PBS evolved independently from each other according to necessities of cyanobacteria under different stress conditions (like high light condition, low light condition, UV light, high temperature, etc.) (Six et al., 2007[86]). During the course of evolution, cyanobacteria appear to have acquired more and more sophisticated light-harvesting complexes, from simple C-PC rods to elaborate rod structure comprising three distinct PBP types *viz* PC, PE I and PE II (Six et al., 2007[85]). On the basis of amino acid sequences as well as the identity and similarities in protein folds, it was suggested that the PBP scaffold evolved from an ancient globin family of proteins (Pastore and Lesk, 1990[73]; Apt et al., 1995[4]).

All PBPs can be traced to a most recent common ancestor protein, which diverged, duplicated, fused and mutated during the development. Apt et al. (1995[4]) have provided a detailed analysis on sequence comparison and phylogenetics, which suggests that at least three steps of gene duplication may have occurred during the evolution of PBPs (Apt et al., 1995[4]). First, a duplication of the ancestral gene generated a pair of tandem PBP genes. Second, this heterodimer gene gave rise to two separate lines of descent through duplication, generating the core (AP) and the rod PBPs. Third, the rod precursor duplicated again to form the PC and PE subfamilies. Core of PBS i.e., AP evolved long before the evolution of PE and PC, quite probably together with the ancestral genome of cyanobacteria (Zhao and Qin, 2006[104]). It implies that light-energy transfer from the PBS core to PSII is an evolutionary, ancient and conservative mechanism that has not allowed much phenotypic variability during the course of evolution (Six et al., 2007[85]). In contrast, rod region appears to have evolved later through complex episode of gene duplication, horizontal gene transfer or gene acquisition from cyanophage. It suggests that the process of light-energy transfer from PBS rods to PBS cores evolved according to necessities of bacteria under different stress condition. In rod region, the phycocyanin gene seems to have evolved before the phycoerythrin gene (Six et al., 2007[85]). This evolutionary adaptation has led to the development of a modular and flexible antenna system that can acclimate to a wide range of environmental conditions to optimize photosynthesis (Neilson and Durnford, 2010[68]). Koziol et al. (2007[50]) proposed LHC evolutionary studies of photosynthetic organisms in great details. The gene cluster for rods, in blue green strain lacking PE, is mainly composed of two identical *cpcB-A* operons encoding the C-PC α- and ß-subunits and gene encoding three rod linkers, one rod core linker and two types of phycobilin lyase (Zhao et al., 2000[105]). Whereas a part of these PC gene cluster is found to be replaced in PE containing strain by a set of 19 genes, likely involved in synthesis and regulation of PE I like complex. This PE region can also be found in all PE II containing strain, but it is interrupted by additional sub region containing 5 to 9 genes, likely involved in synthesis and regulation of PE II complex (Six et al., 2007[86]). On the other hand, the fine tuning of chromophore also plays a significant role in the evolution as well as function of PBPs. Methylation of chromophorylated ß subunit monomer of PC at Asn72 position restricts the conformational flexibility of the Cys82 chromophore and gives the rigid and extended conformation. This change in conformation increases the photosynthetic efficiency by decreasing the excited state proton transfer reactions and intersystem photoisomerization (Zhao et al., 2000[105]). Since Asn72 is very close to the surface of the protein, methylation of Asn72 also restricts the approach of surrounding solvents and oxygen to the chromophore i.e., photo-oxidation in presence of very high light intensity (Miller et al., 2008[63]).

### Atomic resolution structure and evolution of phycobilisome

**Figure 4:**
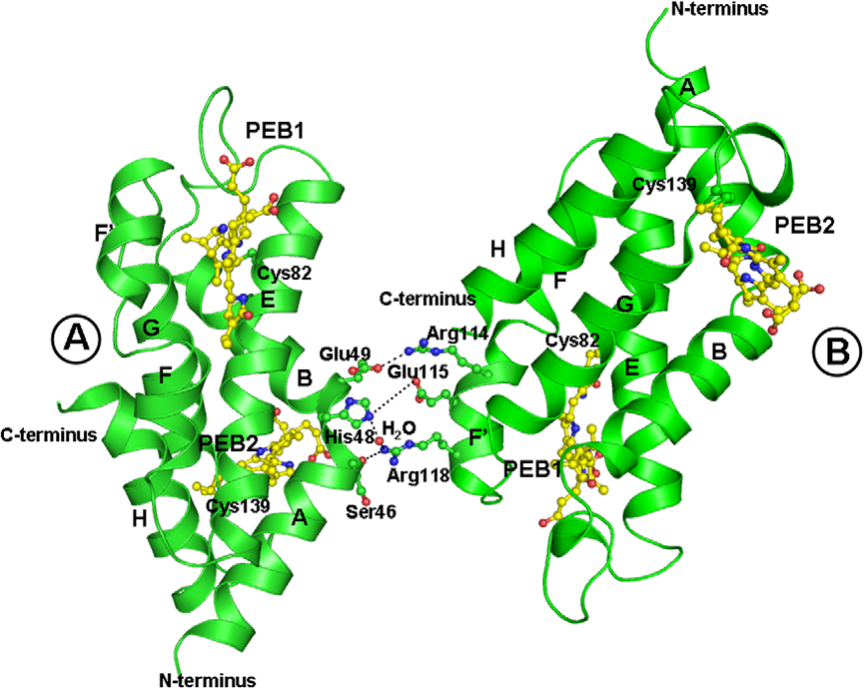
Interaction between two crystallographically individual molecules of F-αPE; A and B is shown by ribbon diagram constructed using PYMOL. The interaction between molecule A and B is indicated by the dotted lines. A and B molecules were found to be interactive via two ionic interactions, two ionic bonds and few van der Waals contacts.

The basic “repeating unit” of the PBS is PBP consisting of two related but distinct polypeptide chains of α and ß. The α- and ß-subunits are made up of six alpha-helices each. These six alpha-helices (A, B, E, F, F’ and H) form a globular core of phycobiliproteins, plus there are two additional helices (X, Y) that extend from the core and they are necessary for assimilation. The heterodimers of α and ß are assemble into (α_3_ß_3_) trimer and two such trimmers constitute an (α_3_ß_3_)_2_ hexamer. Structural studies suggest that Asp13 of α-subunit plays an important role in the formation of stable α-ß dimer. Furthermore, Asp13 also plays a role in preventing ß-ß homodimerization by destabilizing the interaction of the N-termini of identical ß-subunits (Soni et al., 2010[90]). The positive charge at the N-terminus of the α-subunits may similarly be unfavorable for α–α association. However, two α-subunits in truncated conditions are reported to interact via two ionic interactions and two hydrogen bonds together with a few van der Waals contacts (Figure 4(Fig. 4)) (Soni et al, 2010[90]). The structure of PC (PDB ID: 1CPC, *Fremyella diplosiphon*) is composed of a helices connected via ß-turns and has a globin like fold. The monomeric unit has three chromophores attached at α-Cys84, ß-Cys84 and ß-Cys155 that are responsible for the spectral properties of PC (Table 1(Tab. 1)). The trimeric organization of PC brings the chromophore linked to ß-Cys84 of monomer in close proximity (~21Å) to the α-Cys84 chromophore of the neighboring monomer (Duerring et al., 1991[18]). The ß-Cys84 chromophore is oriented into the central cavity of the disc while the ß-Cys155 linked chromophores are located at the periphery of the trimer. In this organizational scheme, the inter-chromophore distances are of the order of 20-50 Å, which are too large for effective excitonic coupling and energy transfer may be accomplished by Forster type of interaction (Soni et al., 2010[90]). Inter-trimer energy-transfer is attributed to ß-Cys155 chromophores while inter-hexamer energy-transfer occurs via chromophores attached to ß-Cys84 positions. The structures of PEC share several features with PC structures (Ficner et al., 1992[22]) except one of the attached bilins. The alpha-subunit of PEC contains a phycobiliviolin (PVB) chromophore with a pi-conjugation that gives it unique spectral properties.

The overall structure of PE bears resemblance to PC and has conserved globin fold (Duerring et al., 1990[17]) except the attached bilin group: chromophores attached at α-Cys82, α-Cys139 and ß-Cys50/61, ß-Cys84, ß-Cys155. Wilk et al. (1999[102]) crystallized and defined the structure of cryptophytes PE having ß-chain structure is similar to α- and ß-chains of other known PBPs. But the overall structure of PE 545 is novel with a chain forming a simple extended fold with an antiparallel ß-ribbon followed by an α-helix (Wilk et al., 1999[102]). AP shares modest sequence similarity with the other subunits of the PBS i.e., PC and PE, but shows a greater degree of structural conservation as the overall fold of the molecule is similar to that of PC and PE (Apt et al., 1995[4]). Both α- and ß-subunit of AP bound to one bilin each (Table 1(Tab. 1)). The α-PCB of AP has a different environment as compared to PC and it has been suggested that this is responsible for the spectral shift of AP. Bryant et al. (1985[12]) described the physiological significance and existence of AP hexamer or trimer (Liu et al., 1999[54]) in details. The α-ß monomer is assembled to form (α_3_ß_3_) trimer and further (α_3_ß_3_)_2_ hexamer. Hexameric assembly of AP is found to be less well-packed than that of PC and PE (Bryant et al., 1979[11]). Loosely-packed AP core has high flexibility, which allows the possible mechanism of interaction between the AP cores and rods. The interactions that stabilize the AP hexamer are also different in two crystal structures. In PDB 1ALL, the ß-subunits mediate the formation of an AP hexamer while in PDB 1KN1, the inter-monomer interface is formed by the α-subunits.

### Complementary Chromatic Adaptation (CCA)

**Figure 5:**
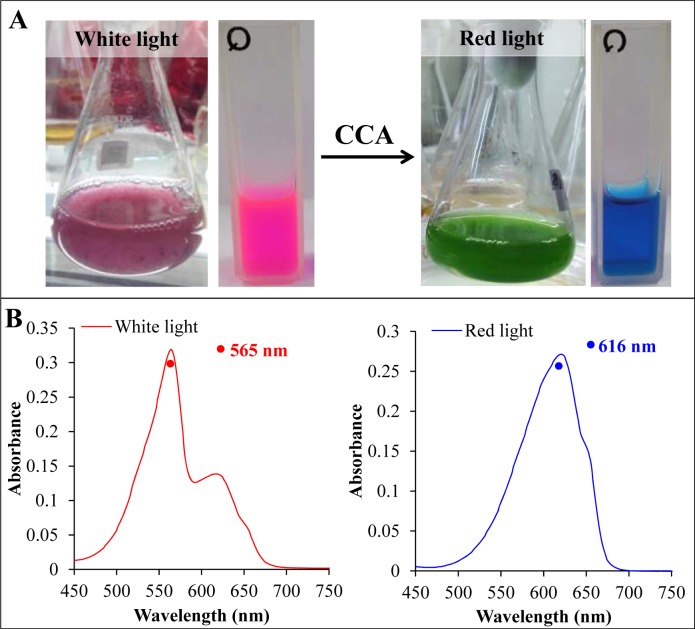
(A) The cyanobacterium *Lyngbya* sp. A09DM, grown in liquid media and fully acclimated to white (left) and red light (right). The color of crude extract (shown along with cell mass) indicated the alteration in phycobilisome pigment composition in a complement manner to absorb the available light. (B) The absorbance spectra of crude extract of white and red light acclimated *Lyngbya* sp. A09DM. Red light exposure switches the dominance of phycoerythrin by phycocyanin in phycobilisomes.

Many, but not all, cyanobacterial species are capable of acclimating to changes in ambient light quality by incorporating several different PBSs and linker proteins into their PBP. This process, known as complementary chromatic adaptation (CCA) (Kehoe and Grossman, 1994[43]) (Figure 5(Fig. 5)), increases photosynthetic efficiency under a range of light conditions by allowing the organism to precisely tailor its light-harvesting pigment composition to closely match the ambient light spectrum. Some thermotolerant cyanobacteria also exhibit change of pigmentation in response to temperature (Singh et al., 2012[84]). The molecular principles that underlie such molecular adaptations are only partly understood. But preliminary investigations suggest extensive inactivation of chromophore protein turn over, by which chromophore conformation and dynamics are modulated (Miller et al., 2008[63]). The CCA is grouped on the basis of PC and PE content of chromatically adapting cyanobacterial species in red and green light respectively (Kehoe, 2010)[42] into following categories:

*Group I* species have unaltered PC and PE levels during growth in red/green light conditions and comprise 27 % of total cyanobacterial species e.g., Red alga.

*Group II* species have PE levels that are high in green light and low in red light, whereas PC levels are not affected by light colour and comprise 16 % of total cyanobacterial species e.g., *Nostoc punctiforne*.

The most complex *Group III *species comprise 57 % of total cyanobacterial species; they also have PE levels that are high in green light and low in red light, like group II e.g.,* F. diplosiphon*. However, additional regulation is exercised in PC content in response to light color-PC levels are high in red light and low in green light, which contrasts PE regulation.

*Group IV* was discovered more recently and is distinct from group I-III, as PC and PE do not change; instead, a green-light absorbing bilin is added to PE in green light and a blue-light-absorbing bilin is added to PE in blue light (Kehoe, 2010[42]) e.g., *Synechococcus* strain.

Given the fairly ubiquitous distribution of the property of light harvesting, it is remarkable that although red macro algae use PBS for light-harvesting, none are known to undergo CCA (Kehoe and Gutu, 2006[44]). CCA is the massive reconstruction of the photosynthetic light-harvesting antennae, or PBSs, which are responsible for providing light energy primarily to photosystem II reaction centers (Kehoe and Gutu, 2006[44]).

Chromatic adaptation is a result of changes at the level of specific gene expression. It includes biosynthesis of light-harvesting apoproteins, several unidentified membrane-associated soluble proteins and chromophore (Stowe-Evans et al., 2004[93]). Radio-isotopic pulse-chase experiments demonstrate that chromatic adaptation does not involve degradation of pre-existing PBSs (Glazer, 1977[29]). When phycoerythrin synthesis is halted by transfer of a culture to red light, the content of this pigment is decreased per cell by cell multiplication and cell death. Change in pigmentation is achieved through the induction of hormogonia formation, which are resistant to adverse environmental condition. Regulation of CCA is based on two distinct mechanisms: (a) Redox control and (b) Photoreceptor control. Regulation through redox state of photosynthetic electron transport chain is controlled by oxidation/reduction of plastoquinone (PQ) (Glazer, 1977[29]). In both red/green lights, PQ-pool redox poise favors oxidation which in turn reduces photosynthesis efficiency by 40 %. The transient reduction in photosynthetic efficiency immediately after a shift in light color (red/green) lasts for ~ 48 hours and is known as “acclimation state”. Once the PBS composition is optimized for absorbance of the ambient light colour, the photosynthetic activity gets recovered and PQ retains its reduced state, called “steady state”. Therefore, the long-term CCA responses that persist during the steady state would be predominantly photoreceptor-controlled. Analysis of photoreceptor-controlled mechanism leads to the identification of light dependent regulator for complementary chromatic adaptation (Rca), CcaSR and control of green light induction (Cgi) systems which regulate PBP synthesis during CCA (Gutu and Kehoe, 2012[31]; Hirose et al., 2013[33]; Rockwell et al., 2012[76]). These systems asymmetrically controlled the expression of PE and PC gene operon. The Rca system comprised of different regulatory proteins designated as Rca family proteins, encoded by *rca* gene cluster. It is a complex photoreceptor based two-component regulatory system, of which the receptor, Rca E, contains histidine kinase domain and chromophore binding domain at C-terminal and N-terminal, respectively. Rca E senses the variation in light wavelength and initiates the Rca cascade by conveying the message to the response regulators. It either phosphorylates or dephosphorylates the downstream element of Rca system. Whereas response regulators are transcriptional controlling elements which further govern PE and PC operon expression. Rca F and Rca C proteins are such response regulators of Rca system, which are substrates for Rca E. A series of experiments suggested that the predominantly phosphorylated state of the Rca pathway in red light results in green phenotype. However non-phosphorylated state in green light results in blue-green phenotype (Kehoe and Gutu, 2006[44]).

Till date, very few components of Cgi and CcaSR systems have been identified or isolated, although this might be expected given that it controls steady-state CCA responses. Gutu and Kehoe (2012[31]) also proposed that CCA in Group II is regulated only by utilizing Cgi and CcaSR systems whereas in Group III, CCA is controlled by Rca and Cgi systems (Gutu and Kehoe, 2012[31]). So further study of Cgi pathway may help to establish whether Group II strains were derived from Group III by loss of the Rca system, or if group III strains arose from group II strains through the acquisition of Rca pathway, or if both events occurred (Gutu and Kehoe, 2012[31]). Rca system and its interaction with Cgi system is discussed in great detail by Kehoe and Gutu (2006[44]).

### Bilin

Bilins are open chain tetrapyrroles covalently attached to the cysteine residue of apoprotein via at least one thioether linkage, sometimes two (Glazer, 1989[27]), and serve as the site for fluorescence origin. In cyanobacteria, up to four bilin chromophores are post-translationally attached to specific cysteines of as many as a dozen or even more individual proteins (Scheer and Zhao, 2008[80]). Apart from phycocyanobilin and phycoerythrobilin, there are two other types of bilin found on certain biliproteins in the cyanobacteria, phycourobilins and phycoviolobilin (Samsonoff and MacColl, 2001[77]), which allow better light-harvesting in particular regions of the spectra. The diverse colours generated in this simple manner cover almost the total range of visible wavelengths from 495 nm for phycourobilin (PUB) to 650 nm for phycocyanobilin (PCB).

#### Properties and biosynthesis of bilins

In heterotrophs, the formation of open chain tetrapyrrole products is considered a catabolic process and it is often related to iron acquisition from heme. Bilin biosynthesis in phototrophs, on the other hand, may be classified as an anabolic pathway as bilins function as chromophore and protein cofactor with light-harvesting (PBSs) and/or light-sensing (phytochromes) properties (Dammeyer and Frankenberg-Dinkel, 2008[14]). The numbers and types of chromophores associated with a particular type of PBP subunit are usually invariant, but are attached through the cysteinyl residue of protein. Free bilin (and bilin in denatured PBSs) fluoresces very weakly due to the preferred cyclohelical conformation in solution. This indicates that the excitation energy is lost rapidly by radiation-less relaxation pathways from free bilins and bilin associated with non-native protein environment. The chemistry that is responsible for special spectral features of bilins is variations in the length of conjugated double bond system, which allows light absorption at different wavelengths. The bilin in PBPs are held rigidly in extended conformation (reduced freedom of molecules) which makes the excited state life-time longer for efficient energy transduction between proteins. The spectroscopic properties of each bilin within PBS are strongly influenced by the conjugation and environment imposed on the bilin by the native protein. Inter-αß interaction makes an important spectroscopic contribution. For example, monomeric (αß) allophycocyanin has λmax at 615 nm whereas λmax of (αß)_3_ lies at 650 nm. This intermolecular energy transfer within PBP trimer and hexamer is very fast and the steady state fluorescence emission originates in consequence. Crystallographic and spectroscopic studies on numerous PBPs have led to the identification of donor and acceptor bilin in C-phycocyanin (PDB ID: 1CPC, *Fremyella diplosiphon*) as well as in other PBPs. For example, in C-phycocyanin the bilin at Cysα-84 and Cysß-155 are donor (lie towards periphery) and the bilin at Cysß-84 (extended in to center) is the acceptor (Dammeyer and Frankenberg-Dinkel, 2008[14]).

**Figure 6:**
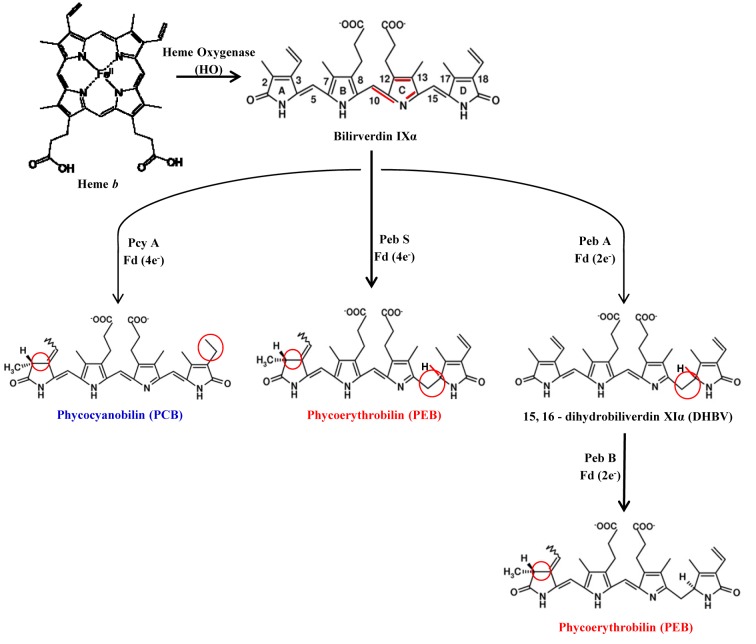
The biosynthetic pathway of phycobilins. Bilin synthesis starts from the cleavage of heme b (bilin b) by hemeoxygenase (HO) to yield biliverdin IXa, the central molecule of bilin biosysnthesis pathways. Biliverdin IXα is further reduced by either NAD(P)H- or ferredoxin-dependent bilin reductases (FDBR). FDBR reductase like PebA synthase and PebB synthase transfer two electrons to their substrate to give phycoerythrobilin in two step catalysis. PcyA and PebS catalyze the transfer of four electrons from ferredoxin to two distinct double bonds of biliverdin to yield phycocyanobilin and phycoerythrobilin, respectively in single step. Phycocyanobilin and phycoerythrobilin are further reduced to phycoviobilin and phycourobilin, respectively by PecE/F of Rpc G reductase.

Bilin is widely distributed in all kingdoms of life except archaea and serves distinct functions. The initial step in the biosynthesis of functional open chain tetrapyrroles is catalyzed by an enzyme hemeoxygenase (HO) where heme serves as both the substrate and cofactor for its degradation. All characterized HOs from photosynthetic organisms cleave the heme, regiospecifically at the α-*meso *carbon resulting in the IXα isomer of biliverdin. Bilirubin and phytochrome are synthesized by reduction of biliverdin IXα catalyzed by enzyme biliverdin reductase (BvdR) and phytochromobilin (PϕB) synthase using NADH and ferrodoxin as proton donour respectively. Bilin biosynthesis from biliverdin IXα is catalyzed by feredoxin dependent bilin reductase (FDBR) enzyme. Phycocyanobilin and phycoerythrobilin are synthesized in two successive reductions by ferrodoxin dependent enzymes PcyA and PebA/PebB respectively (Dammeyer and Frankenberg-Dinkel, 2008[14]) (Figure 6(Fig. 6)).

### Chromophorylation of phycobiliproteins

Attachment of cofactor (bilin) to PBP and phytochrome like receptor is lyase mediated and autocatalytic respectively (Scheer and Zhao, 2008[80]). Although, spontaneous attachment of chromophore to PBS with low fidelity* in vitro* has been reported (Fairchild and Glazer, 1994[20]). Various lyases are required for chromophore attachment to specific conserved cysteine residues of the protein and yields only one attachment product (Soni et al., 2008[91]). To date, four types of lyases have been described: (1) E/F-type lyases, (2) S(U)-type lyases and (3) T-type lyases (4) Y/Z-type lyase (Soni et al., 2008[91]). The E/F-lyases are heterodimeric and are required for correct binding of chromophore to Cysα-84 of the PBS (Zhou et al., 1992[107]; Fairchild and Glazer, 1994[20]). Nomenclature of different E/F-lyases proposed by Wilbanks and Glazer (1993[101]) and others depends basically on the kind of bilin whose attachment is catalyzed by the lyase. It is of interest to note that E/F-lyases may also catalyze both release and isomerisation of attached chromophores (phycocyanobilin to phycoviobilin, and phycoerythrobilin to phycourobilin). S-type lyases are universal in distribution, and have an even larger spectrum of activity. They may form hetero-dimers with V-type lyases. S-type lyases catalyze binding of chromophores to α- and ß-subunits of phycocyanin and phycoerythrin, via Cys84. T-type lyases, on the other hand, specifically catalyze the binding of PCB at cysteine 153 ß-subunits of C-PC (Zhao et al., 2007[106]). Y/Z-type lyases, also known as PE-I and PE-II lyases, are hetrodimeric and catalyze the binding of PEB to PE α- and ß-subunits, but the precise site specificity of this enzyme is still unknown (Scheer and Zhao, 2008[80]). Moreover, Shukla et al. (2012[81]) have identified the isomerase Mpe Z with lyase activity in marine *Synechococcus*, attaches phycoerythrobilin to cysteine-83 of the α-subunit of phycoerythrin II and isomerizes it to phycourobilin upon green to blue light shifting (Shukla et al., 2012[81]).

It is significant to note that *in vitro* studies performed by Kupka et al. (2009[52]), have suggested that lyses may function as chaperons, which binds the bilin chromophore to the appropriate apo-protein in correct conformation and stereochemistry to avoid synthesis of unwanted by-products. 

### Linker polypeptides

Linkers are nonchromophoric basic polypeptides which are hydrophobic in nature and join the consecutive PBPs. Linkers are involved in face-to-face aggregation of PE, PC and PEC trimers that are involved in higher order assembly of the PBS. The process is mediated by tail-to-tail joining of hexameric assemblies into cylinders of rods and core. The linker itself is buried in the central cavity of PBP discs by the combination of hydrophobic and multiple charged interactions (Sidler, 1994[82]), and thereby plays important roles in maintaining PBS structure and energy transfer (Tandeau de Marsac and Cohen-Bazire, 1977[96]; Sidler, 1994[82]). However, the PBSs would not be able to arrange themselves in the configuration necessary for efficient energy transfer without linker proteins or peptides. Glazer (1985[27]) provided a widely used classification system which defines linker polypeptides with respect to their location and molecular masses (Glazer, 1985[27]) represented as subscript and superscript respectively. Using this classification system, linker polypeptides can be divided into four groups according to their functions and locations in the PBS: Group I, L_R_ polypeptides (MW: 27 to 35 kDa, including small rod linkers of 10 kDa), participates in the assembly of the peripheral rod substructure and link PE/PC trimers to hexamers or PE/PC hexamers to other PE/PC hexamers; Group II, L_RC_ polypeptides (MW: 25 to 27 kDa), is involved in attaching the peripheral rods to the AP cores and may function in the assembly of rod substructure; Group III, L_C_ polypeptides (MW: 7 to 8 kDa) is a portion of core components and plays key roles in the assembly of the core substructure, and Group IV, L_CM_ (MW: 70 to 120 kDa), also known as ApcE or anchor polypeptide, is involved in the attachment of the PBSs to the membrane, and is envisaged as one type of terminal acceptors of excitation energy within the PBSs (de Lorimier et al., 1990[16]). ApcE plays a vital role in interaction of PBS with the membrane as a proportion of ApcE remains membrane-associated even after all the other PBPs have been washed away (Redlinger and Gantt, 1982[75]). ApcE has multiple large domains with similarity to rod linker sequences that may support AP hexamer formation (Houmard et al., 1990[34]). ApcE (L_cm_) also contains a chromophore, as well as some gamma subunits of PE that are phylogenetically linker proteins. To understand the mechanism of assembly and function of linker polypeptides, several attempts have been made to isolate different linker polypeptide to its pure form. However, due to its high hydrophobicity and their high susceptibility to proteolytic degradation, this purification is difficult and gives very low yields. For this reason, linker proteins are mostly studied in PBP-linker complexes (Fuglistaller et al., 1986[24]). The principle for isolation of linker polypeptides from intact PBS is their basicity (*pI* > 7.0) as compared to acidic (*pI* < 5.8) PBPs. Additionally, linker isolation and purification by gel-permeation and reverse-phase chromatography has been reported by Fuglistaller et al. (1986[24]).

Alignment results indicate that the primary sequences of linkers are less conserved than those of their associated PBP subunits. Among all linker polypeptide, L_C_ shows a comparatively high level of sequence identity in different algae, indicating possible conservation among ancestral linker polypeptides. In case of L_R_ polypeptide, many conserved domains near the N-terminus are presumed to play crucial roles in packing into a central channel of the PBP hexamers, whereas other regions may provide the assembly interface between rod discs (Sidler, 1994[82]). L_RC_ linkers are more distantly related to the rod linker polypeptides. However, six conserved domains (22-kDa fragment) have been identified within the N-terminal of these linker proteins, which were presumed to occupy the central hole of (αß)_3_^PC^ and are largely protected in proteolytic treatment. Based on the sequence analysis and proteo-lysis experiments of linker polypeptides, Parbel and Scheer (2000[71]) proposed an interlocking model for the PBS rod organization in which the linker polypeptides in the model are proposed to possess two distinctive domains. The N-terminal domain is hypothesized to be buried in the central hole of the trimer and protected from proteolysis, whereas the C-terminal domain is believed to protrude from the hexamer. Kondo et al. (2007[49]) found that *Synechocystis* sp. PCC 6803 possesses two types of PBSs that differ in their interconnecting ‘‘rod-core linker’’ proteins (CpcG1 and CpcG2) (Kondo et al., 2007[49]). CpcG1-PBS was found to be equivalent to conventional PBS, whereas CpcG2-PBS retains phycocyanin rods but is devoid of the central core. Sequence analysis revealed that CpcG2-like proteins containing a C-terminal hydrophobic segment are widely distributed in many cyanobacteria. Recently Marx and Adir (2013[61]) found that linker protein actually stabilizes the interaction of rods and core cylinders in higher assembly (David et al., 2011[15]; Marx and Adir, 2013[61]).

Future studies on structure and function of linker polypeptides will require more effective approach that will allow their study in solution in the absence of PBP.

### Organization of phycobilisome on stromal membrane and energy transfer

PSI and PSII are simultaneously present on the thylakoid membrane and provide the site for attachment of PBS. Low-temperature fluorescence spectra of phycobilin excitation indicate that a significant proportion of PBS-absorbed energy is delivered to PSII as well as PSI (Ashby and Mullineaux, 1999[6]). Recent finding of Liu et al. (2013[53]) suggests no physical interactions between the PBS core and PSII and I are required for energy transfer. However the cytochrome b_6_f complex, which connects PSI and PSII by mobile-electron carriers to complete the electron transport chain. Kirilovsky and Kerfeld (2013[48]) reviewed and suggested the involvement of orange carotenoid protein (OCP), in the energy transfer between PSII and PSI. Presently it is unclear whether binding of PBS to PS I is mediated by specific interactions or by transient association (Aspinwall et al., 2004[7]). Indeed, direct measurements of fluorescence recovery using confocal microscopy has indicated that the diffusion of PBS is quite rapidly as compared to photosystems (Mullineaux et al., 1997[67]), indicating that the association between antenna and photosystems in cyanobacteria may be much looser than in other systems (Adir, 2005[1]). However the PBS diffusion is generally slower in comparison to similarly sized proteins from other eukaryotic membranes or organelles (Kana, 2013[38]). On the other hand, an early study showed that PBSs are structurally associated with PSII only, and that energy is transferred onwards from PSII to PSI by “spillover” or excitation exchange between the chlorophylls of the PSII and PSI core complexes rather than the change in association of the PBSs to PSII (mobile antenna model). Vernotte et al. (1990[98]) proposed that the excitation exchange between PSII and PSI unit depends on the mutual orientation of photosystems rather than their distance. However, existing evidence indicates that spillover is certainly not the only route for energy transfer to PSI (Mullineaux, 1994[65]). Another possibility is that PBS core can interact directly with PSI much in the same way that it is thought to interact with PSII (Su et al., 1992[94]). Alternatively, there might be a direct interaction of PBS rods with PSI, allowing an alternative energy transfer pathway by-passing the PBS core (Su et al., 1992[94]). However in high light condition, orange carotenoid protein (OCP) has found to directly interact with PBS to trigger photoprotective mechanism via increasing thermal dissipation of excess absorbed energy (Kirilovsky, 2010[46]; Kana et al., 2012[39]). Coupling and uncoupling of OCP-PBS complex is regulated by 14-kDa thalakoid membrane protein called as fluorescence recovery protein (FRP). FRP greatly accelerates the conversion of the active red OCP (attached to PBS) into the inactive orange OCP (free form) in low irradiation, maintaining the orange OCP pool (Boulay et al., 2010[10]).

It was believed earlier that direct association of the PBSs with lipid bilayer also takes place with the help of “ApcE” due to the presence of membrane interacting domain. Nevertheless, analysis of amino acid sequence of ApcE did not reveal any obvious membrane-integral domains (Houmard et al., 1990[34]). Furthermore, deletion of PB-loop from ApcE did not affect the interaction of PBSs with the lipid membrane (Ajlani and Vernotte, 1998[2]). Study of PS mutant *Synechocystis* revealed that PBSs are assembled normally and are membrane-associated even when no PSII or PSI reaction centers are present (Glazer, 1985[27]). This observation suggests a direct association of the PBSs with the lipid bilayer. Diffusion measurement experiments below phase transition temperature showed decline in membrane lipids diffusion (Sarcina et al., 2001[79]), whereas PBS diffusion is not affected (Sarcina et al., 2003[78]). This suggests an absence of integral membrane domain in PBS, but instead interacts with lipid head-groups at the membrane surface. For efficient transfer and distribution of energy to the reaction centers, PBS of several cyanobacterial strains also contains one to two molecules of enzyme ferredoxin NADP^+^ oxidoreductase (FNR). It has been proposed that FNR is situated at the interface of the two lower PC rods closest to the thalakoid membrane and catalyzes the electron transfer between ferredoxin and NADP (Lüder et al., 2001[56]).

Energy transfer and kinetics of PBS-RC complex at room temperature was measured by picoseconds time-resolved fluorescence. A relatively short and long overall lifetime suggests the presence of coupled and decoupled PBS-RC complex. Decoupled PBSs diffuse rapidly on the membrane surface, but are immobilized when cells are immersed in high-osmotic strength buffers, apparently due to stabilized interaction between PBSs and RC is stabilized (Yang et al., 2007[103]). This suggests a dynamic model in which each individual PBS is coupled to a RC most of the time, but always with a strong probability of decoupling. Decoupling of PBS from a RC is followed by a brief period of rapid diffusion before re-attachment to another reaction center, where the diffusion period depends on density of reaction centers (Sarcina et al., 2003[78]). Mullineaux (2008[66]) and Kana (2013[38]) have very nicely reviewed the numbers regarding the diffusion velocity and fractions decoupled PBS under normal conditions. 

A gene required for the short-term regulation of photosynthetic light harvesting (the state transition) has been identified in the cyanobacterium *Synechocystis* sp. PCC6803, encodes 16 kDa protein designated as Rpa C (regulator of phycobilisome association C). Moreover the* in vitro* [γ-^32^P]-ATP labeling experiments suggest that Rpa C is not the 15 kDa membrane phosphoprotein previously implicated in state transitions (Emlyn-Jones et al., 1999[19]). Joshua et al. (2005[37]) found the pool of free PBS that are neither functionally nor structurally coupled to reaction centers after prolong iron-starvation (Joshua et al., 2005[37]). On the other hand McConnell et al. (2002[62]) suggested intramembranous transfer of excitation from* Chl a* holochromes of PSII to Chl *a* holochromes of PSI - the so-called spill-over phenomenon instead of direct involvement of PBP diffusion (McConnell et al., 2002[62]). Long distance diffusion of PBS require its decoupling from the membrane surface, which is induced either by excessive irradiance or by short-term heat stress; therefore understanding of PBS decoupling mechanism requires additional research (Kana et al., 2009[40]). The PBS decouplings observed under very high and low light are assumed to be a strategy of PBS to prevent photodamage photosynthetic reaction centers (Liu et al., 2008[54]). Some cyanobacteria which lake other photoprotective mechanism also use the state transition (S to M fluorescence transition) as an alternative mechanism for the dissipation of excess energy (Kana et al., 2009[40]). Decoupling of PBSs may serve as an emergency valve for protection against photo-oxidative damage when the protective capacity of all other light-adaptation responses has been exhausted (Tamary et al., 2012[95]). Many attempts have been made to isolate the cyanobacterial thylakoid membranes with functionally coupled PBSs using buffer of very high osmotic (Katoh and Gantt, 1979[41]) and ionic strength (Glazer, 1988[28]) in the presence of detergent. However, Mullineaux (2008[66]) has reviewed that the results of his own experiments are extremely inconsistent and showing very poor energy transfer in above mentioned conditions. This effectively limits our *in vitro* study of cyanobacterial thylakoid membrane with functionally coupled PBSs. 

## Conclusion

Sequential development of light-harvesting antennae complexes in lower photosynthetic organism made energy transduction very efficient. Hydrophilic PBPs are a distinctively coloured group of disk-shaped macromolecular proteins orderly assembled into PBS with the help of linker polypeptides. High-resolution X-ray crystallography coupled with the knowledge of amino acid sequence of proteins such as F-αCPE, (often via gene analysis) has provided a detailed picture of the arrangement and interactions of PBP within PBS. Thus, biologist must begin to think more like engineers and information scientists in order to develop a meaningful understanding of energy transfer in cyanobacteria and understand the complex multimeric PBS.

## Notes

Niraj Kumar Singh and Ravi Raghav Sonani have contributed equally to this work.

Rajesh Prasad Rastogi and Datta Madamwar (e-mail: datta_madamwar@yahoo.com) have both contributed equally as corresponding authors.

## Acknowledgements

NKS gratefully acknowledges the Department of Science and Technology (DST), New Delhi for financial support in the form of DST (SERB) FAST TRACK YOUNG SCIENTIST fellowship. RRS acknowledges DST, New Delhi for financial support in form of INSPIRE fellowship [IF120712]. RPR is thankful to the University Grant Commission (UGC), New Delhi, India, for financial support in the form of Dr. DSK Postdoctoral fellowship. We also thank Er Vijay Mungla for valuable help in drawing the illustrations. The authors also thank Dr. Varun Shah (National Center for Antarctica and Ocean Research (NCAOR), Goa, India, for useful discussion and critical suggestions on the manuscript.

## Figures and Tables

**Table 1:**
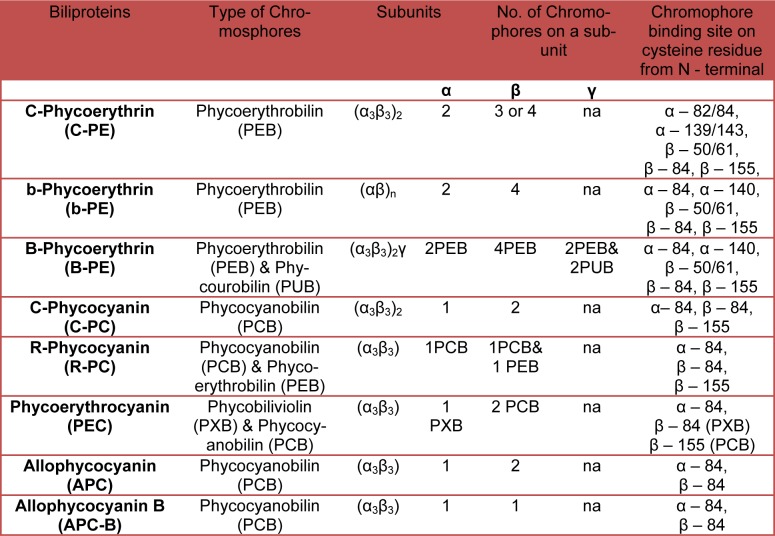
Subunit structure, type, number and location of chromospheres on the Phycobiliprotein
